# Phytochemical Composition and In Vitro Anti-Pigmentation Activity of *Persicaria senticosa* Flower Absolute: Potential Dual Inhibition of Melanogenesis and Melanosome Transport

**DOI:** 10.3390/ph19071129

**Published:** 2026-07-22

**Authors:** Kyung Jong Won, Hwan Myung Lee, Yoon Yi Kim, Ji Hye Bae, Ji Seong Yun, Do Yoon Kim

**Affiliations:** 1 Department of Physiology and Premedical Science, College of Medicine, Konkuk University, Chungju 27478, Republic of Korea; kjwon@kku.ac.kr; 2 Department of Biotechnology, College of Bio-Health, Hoseo University, Asan 31499, Republic of Korea; kacsital@hoseo.edu (H.M.L.); yoontwo22@gmail.com (Y.Y.K.); bjh001128@naver.com (J.H.B.); dbswltjd001@naver.com (J.S.Y.); 3 Korea Essential Oil Resource Research Institute, Hoseo University, Asan 31499, Republic of Korea

**Keywords:** *Persicaria senticosa*, flower absolute, melanogenesis, melanosome transport, B16BL6 cells, anti-pigmentation, skin lightening

## Abstract

**Background/Objectives**: *Persicaria senticosa* (Meisn.) H.Gross (PS) has anti-photoaging, anti-inflammatory, and antioxidant activities, but the anti-pigmentation potential of the PS flower absolute (PSFAb) remains largely unexplored. This study aimed to examine the chemical composition and anti-melanogenic and melanosome transport-inhibitory effects of PSFAb using B16BL6 murine melanoma cells. **Methods**: PSFAb was extracted with hexane and analyzed by gas chromatography–mass spectrometry (GC-MS). The biological activities in B16BL6 murine melanoma cells were evaluated using water-soluble tetrazolium salt (WST) assays, 5-bromo-2′-deoxyuridine (BrdU) incorporation, enzyme-linked immunosorbent assays, and immunoblotting methods. **Results**: GC-MS analysis identified eight constituents in PSFAb. Cell viability was not significantly altered in B16BL6 cells at concentrations ≤ 100 μg/mL, which were used for additional tests. PSFAb inhibited serum-induced cell proliferation and suppressed α-melanocyte-stimulating hormone (α-MSH)-induced melanin synthesis and tyrosinase activity in B16BL6 cells. PSFAb also downregulated the α-MSH-induced expression of key melanogenic regulators, including microphthalmia-associated transcription factor (MITF), tyrosinase, tyrosinase-related protein-1 (TRP-1), and TRP-2. PSFAb decreased extracellular signal-regulated kinase 1/2 and p38 mitogen-activated protein kinase phosphorylation but enhanced JNK phosphorylation in α-MSH-stimulated B16BL6 cells. Furthermore, PSFAb reduced the α-MSH-induced expression of melanosome transport-related proteins (melanophilin and Rab27a) in B16BL6 cells. **Conclusions**: Overall, these results suggest that PSFAb has the potential to exert anti-pigmentation effects by suppressing melanogenesis and downregulating melanosome transport-related proteins. Therefore, PSFAb may be a promising candidate for the development of natural agents targeting hyperpigmentation and skin pigmentation regulation.

## 1. Introduction

Skin pigmentation is an important physiological phenomenon determined by melanogenesis. Melanin is not just the pigment that determines skin color; it also plays a key role in protecting the skin from external stimuli such as ultraviolet (UV) radiation [1,2]. In particular, melanin reduces oxidative damage by attenuating UV rays and eliminating reactive oxygen species (ROS), and it helps lower the risk of developing various skin diseases, including skin cancer [1,3]. Nevertheless, melanin may accumulate excessively or abnormally when normal melanogenesis regulation is disrupted, resulting in hyperpigmentation conditions, such as spots, freckles, age spots, and post-inflammatory pigmentation (PIH) [4,5]. These pigmentary conditions are difficult to manage, and they can also adversely affect individuals’ quality of life [6,7,8]. Accordingly, there is growing interest in developing safe, efficient materials that effectively alleviate pigmentation and maintain a uniform skin tone.

Melanin is a complex biochemical product that is synthesized within melanocytes and is regulated by the interactions among various factors, including hormones, genetic backgrounds, UV exposure, and inflammatory responses [6,9]. Among them, microphthalmia-associated transcription factor (MITF) acts as a key regulatory factor and induces melanin synthesis by stimulating the expression of tyrosinase and enzymes such as TRP-1 and TRP-2 [9,10]. In addition, MITF activity is also modulated by the mitogen-activated protein kinase (MAPK) signaling cascades, including extracellular signal-regulated kinase 1/2 (ERK1/2), p38, and c-Jun N-terminal kinases (JNKs), which contribute to melanogenesis [10,11].

On the other hand, skin pigmentation is strongly dependent on melanin production and the transport of melanosomes from melanocytes to surrounding keratinocytes [12,13]. This movement process is mediated by protein complexes consisting of Rab27a, melanophilin, and myosin Va [14,15]. Therefore, strategies for regulating melanin biosynthesis or melanosome transport may be promising approaches for reducing hyperpigmentation and promoting skin whitening.

Natural products have diverse biological activities with relatively few adverse effects, making them attractive candidates for skin whitening or depigmentation [9,10]. Among them, plant-derived essential oils have been widely studied for their roles in health [16,17]. *Persicaria senticosa* (Meisn.) H.Gross (PS) is an annual plant belonging to the Polygonaceae family, which is found throughout East Asia, including Korea [18,19]. PS has traditionally been used in traditional remedies to inhibit inflammation, improve blood circulation, and heal wounds [18,20]. Recent studies have shown that this plant helps prevent photoaging by inhibiting UVB-induced ROS production and collagen degradation and by exerting anti-inflammatory and antioxidant activity [20]. In addition, the PS extract exhibited nitric oxide and elastase inhibitory effects, as well as DPPH and ABTS radical scavenging activities, suggesting that some components have the potential to regulate melanin biosynthesis by inhibiting tyrosinase [19]. Nevertheless, the skin pigmentation regulatory activities of essential oils derived from PS flowers, particularly the flower absolute, in melanocytes are unclear. Therefore, this study examined the effects of the PS flower absolute (PSFAb) on the skin pigmentation-related responses in B16BL6 mouse melanoma cells and analyzed its chemical composition. Our study provides new evidence that PSFAb is associated with reduced melanogenesis and decreased expression of melanogenesis- and melanosome transport-related proteins, supporting its anti-pigmentation potential.

## 2. Results

### 2.1. Gas Chromatography–Mass Spectrometry Analysis of PSFAb Composition

**Figure 1:**
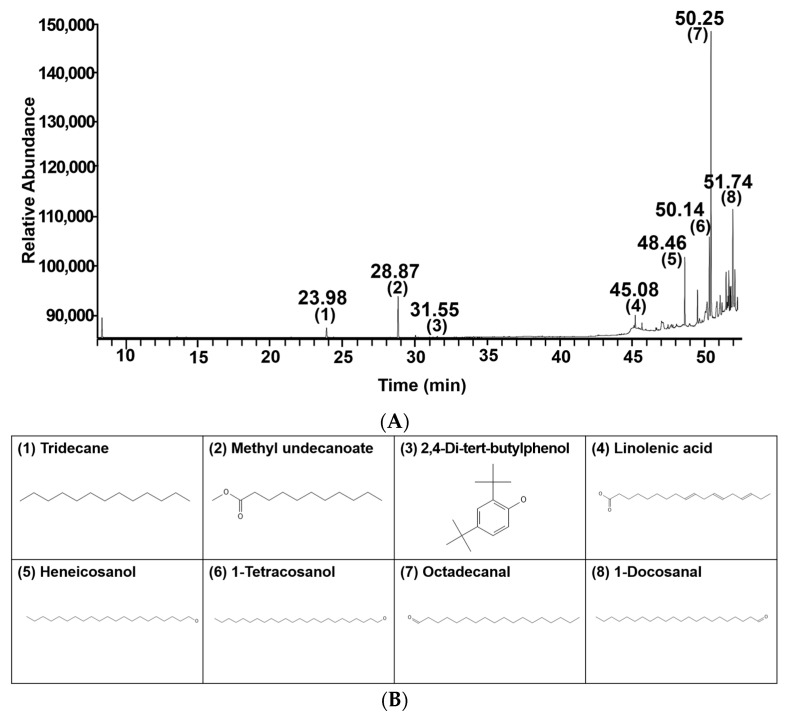
Total ion chromatogram obtained by chromatography–mass spectrometry (GC-MS) analysis of the *Persicaria senticosa* (Meisn.) H.Gross flower absolute (PSFAb). (**A**) The bracketed numbers above each peak correspond to the 8 identified compounds, while the numbers beneath indicate their respective retention times, as detailed in Table 1. (**B**) The structures of eight compounds identified in PSFAb.

The chemical constituents of PSFAb were identified using gas chromatography–mass spectrometry (GC–MS) analysis. GC–MS analysis revealed eight compounds in PSFAb (Figure 1 and Table 1). Among these compounds, 1-docosanal showed the greatest relative abundance, accounting for 41.74% of the total peak area. This was followed by octadecanal (29.51%), 1-tetracosanol (8.21%), linolenic acid (6.90%), heneicosanol (6.47%), methyl undecanoate (5.85%), and tridecane (1.15%). 2,4-di-tert-butylphenol showed the lowest abundance at 0.17% (Table 1). Representative mass spectra of the identified compounds are shown in Supplementary Figure S1. The identified compounds were confirmed based on mass spectral library matching (SI ≥ 700), agreement between experimental and literature retention indices (RIs), and consistency among triplicate GC–MS analyses. The reproducibility and precision of the GC–MS analysis were assessed using the RSD values of RIs for the identified compounds (Table 1).

### 2.2. Changes in B16BL6 Melanoma Cell Viability and Proliferation by PSFAb Stimulation

**Figure 2:**
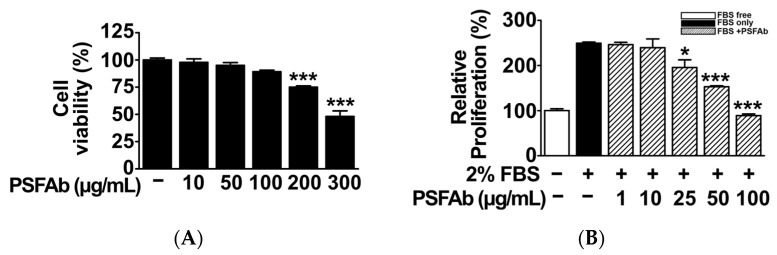
Effects of the *Persicaria senticosa* (Meisn.) H.Gross flower absolute on the viability and proliferation of B16BL6 cells. (**A**) Cell viability. Cells were treated with the *Persicaria senticosa* (Meisn.) H.Gross flower absolute (PSFAb; 10–300 μg/mL) for 24 h, followed by analysis using a water-soluble tetrazolium salt (WST) assay. The experimental groups consisted of the untreated control (−) and PSFAb-treated groups (10, 50, 100, 200, and 300 μg/mL). (**B**) Cell proliferation. Cells were cultured in a minimum essential medium with or without PSFAb (1–100 μg/mL) under conditions containing or lacking 2% fetal bovine serum (FBS) for 48 h. Proliferation was assessed via the 5-bromo-2′-deoxyuridine (BrdU) incorporation assay. In the treatment scheme shown in the figure, “+” and “−” indicate the presence and absence of the indicated treatment, respectively. The experimental groups were as follows: untreated control (without 2% FBS or PSFAb), 2% FBS alone, and 2% FBS plus PSFAb (1, 10, 25, 50, or 100 μg/mL). The untreated control served as the negative control, whereas the 2% FBS-alone group served as the positive control for cell proliferation. The level of the untreated control was set as 100% for viability (**A**) and proliferation (**B**). The results are presented as the mean ± standard error of the mean (SEM) from three independent biological replicates (*n* = 3). Statistical analysis was performed using one-way ANOVA followed by Tukey’s post hoc test. *: *p* < 0.05; ***: *p* < 0.001 compared to the untreated controls (**A**) or cells treated with 2% FBS alone (**B**).

The potential anti-pigmentation-linked biological effects of PSFAb in B16BL6 melanoma cells were evaluated following the assessment of its cytotoxic effects (10–300 μg/mL) using the water-soluble tetrazolium salt (WST) assay. PSFAb (10–100 μg/mL) did not significantly affect B16BL6 cell viability after 24 h of exposure, whereas treatment with 200 and 300 μg/mL significantly reduced cell viability at both time points (Figure 2A). Based on these results, PSFAb was used in a concentration range of 1–100 μg/mL for further evaluation. B16BL6 cell proliferation was then evaluated by 5-bromo-2′-deoxyuridine (BrdU) incorporation assays under 2% fetal bovine serum (FBS)-stimulated conditions to assess the effects of PSFAb. PSFAb at 25–100 μg/mL significantly suppressed B16BL6 cell proliferation in both assays; the strongest inhibitory effects were observed at 100 μg/mL. At this concentration, B16BL6 cell proliferation was 87.22 ± 3.79% of that of the untreated control (Figure 2B).

### 2.3. Alterations in Melanin Production and Tyrosinase Activity in B16BL6 Melanoma Cells by PSFAb Stimulation

**Figure 3:**
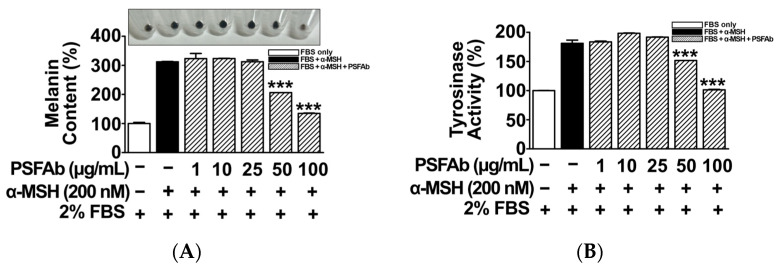
Effects of the *Persicaria senticosa* (Meisn.) H.Gross flower absolute on α-MSH-stimulated melanogenesis and tyrosinase activity in B16BL6 cells. Cells were treated for 48 h with or without the *Persicaria senticosa* (Meisn.) H.Gross flower absolute (PSFAb; 1–100 μg/mL) in the presence or absence of α-melanocyte-stimulating hormone (α-MSH, 200 nM) in the 2% FBS-containing minimum essential medium. In the treatment scheme shown in the figure, “+” and “−” indicate the presence and absence of the indicated treatment, respectively. The experimental groups were as follows: untreated control (2% FBS only), α-MSH alone (200 nM), and α-MSH plus PSFAb (1, 10, 25, 50, or 100 μg/mL). Melanin content (**A**) and tyrosinase activity (**B**) were determined according to the procedures outlined in Section 4. Representative images are shown in the upper panel of (**A**). The untreated control (2% FBS only) served as the negative control, whereas α-MSH (200 nM) alone served as the positive control. The levels of melanin content and tyrosinase activity were normalized to the total protein, and the values for the untreated control (2% FBS only) were set to 100%. Data are presented as the mean ± standard error of the mean (SEM) from three independent biological replicates (*n* = 3). Statistical analysis was performed using one-way ANOVA followed by Tukey’s post hoc test. ***: *p* < 0.001 compared to the cells treated with α-MSH alone.

This study evaluated how PSFAb affects melanin synthesis in B16BL6 melanoma cells. Cells were incubated with PSFAb (1–100 μg/mL) under stimulation with 200 nM α-melanocyte-stimulating hormone (α-MSH). At 50 and 100 μg/mL, PSFAb significantly decreased melanin synthesis stimulated with α-MSH (200 nM), which was increased to 312.89 ± 1.07% compared to the FBS (2%)-alone-treated control group in B16BL6 cells. The strongest inhibitory effect on melanin synthesis was observed at 100 μg/mL, reducing levels to 134.99 ± 1.06% of the FBS (2%)-alone-treated control (Figure 3A). In addition, the effect of PSFAb (1–100 μg/mL) on tyrosinase activity was observed in B16BL6 melanoma cells. Treatment with PSFAb at 50 and 100 μg/mL resulted in a significant suppression of the α-MSH (200 nM)-induced increase in the tyrosinase activity level, which reached 181.08 ± 5.58% of the FBS (2%)-alone-treated control in B16BL6 melanoma cells. The largest decrease in tyrosinase activity was observed at 100 μg/mL, where it decreased to 101.35 ± 0.78% relative to the FBS (2%) control group (Figure 3B).

### 2.4. PSFAb-Induced Changes in Melanogenesis-Related Regulatory Proteins in B16BL6

**Figure 4:**
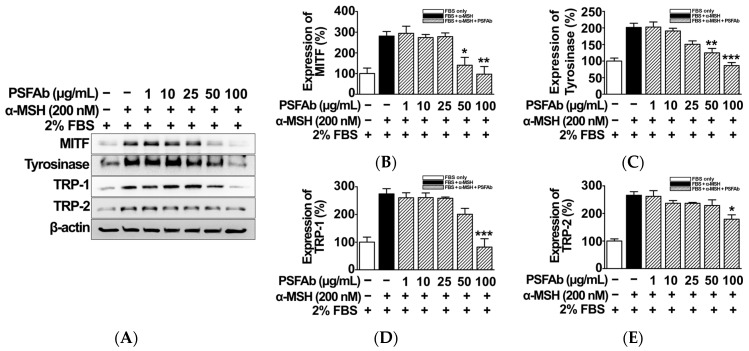
Effects of the *Persicaria senticosa* (Meisn.) H.Gross flower absolute on melanogenesis-related protein expression in B16BL6 cells. (**A**) Representative immunoblot results. The cells were treated for 24 h with or without the *Persicaria senticosa* (Meisn.) H.Gross flower absolute (PSFAb; 1–100 μg/mL) in the presence or absence of α-melanocyte-stimulating hormone (α-MSH, 200 nM) in the 2% FBS-containing minimum essential medium. Protein extracts were analyzed by immunoblotting using the specified antibodies, as described in Section 4. In the treatment scheme shown in the figure, “+” and “−” indicate the presence and absence of the indicated treatment, respectively. The experimental groups were as follows: untreated control (2% FBS only), α-MSH alone (200 nM), and α-MSH plus PSFAb (1, 10, 25, 50, or 100 μg/mL). (**B**–**E**) Statistical graphs resulting from the quantitative analyses of protein expression levels for MITF (**B**), tyrosinase (**C**), TRP-1 (**D**), and TRP-2 (**E**). The untreated control (2% FBS only) served as the negative control, whereas α-MSH (200 nM) alone served as the positive control. The expression levels of MITF, tyrosinase, TRP-1, and TRP-2 were normalized to β-actin as a loading control, and expression levels in the untreated control group (2% FBS only) were set to 100%. The data are presented as the mean ± standard error of the mean (SEM) from three independent biological replicates (*n* = 3). Statistical analysis was performed using one-way ANOVA followed by Tukey’s post hoc test. *: *p* < 0.05; **: *p* < 0.01; ***: *p* < 0.001 compared with cells exposed to α-MSH alone. MITF, microphthalmia-associated transcription factor; TRP-1 and TRP-2, tyrosinase-related protein-1 and -2.

Melanogenesis is regulated by key regulatory proteins, such as MITF, tyrosinase, TRP-1, and TRP-2 [21]. The expression levels of these proteins after treatment with PSFAb (1–100 μg/mL) were examined using Western blot analysis to determine how PSFAb inhibits melanin production in B16BL6 cells. Stimulation with α-MSH (200 nM) increased MITF expression to 281.01 ± 22.23% compared to the FBS (2%)-alone-treated control group. By contrast, PSFAb significantly inhibited this induction at 50 and 100 μg/mL, with the strongest reduction at 100 μg/mL (96.95 ± 36.93% of the FBS (2%)-alone-treated control; Figure 4A,B). Similarly, α-MSH elevated tyrosinase expression to 201.42 ± 12.60% of the FBS (2%)-alone-treated control level, whereas the PSFAb treatment at 50 and 100 μg/mL resulted in a significant reduction in α-MSH-induced expression. The greatest inhibitory effect was observed at 100 μg/mL (86.36 ± 9.17% of FBS (2%)-alone-treated control; Figure 4A,C). In addition, α-MSH (200 nM) increased TRP-1 expression to 274.11 ± 18.80% of the FBS (2%)-alone-treated control. The α-MSH-induced increase in TRP-1 expression in B16BL6 cells showed significant attenuation following treatment with 100 μg/mL PSFAb, reducing the level to 81.14 ± 30.21% of the FBS (2%)-alone-treated control (Figure 4A,D). A similar pattern was observed for TRP-2 expression. Specifically, α-MSH stimulated an increase in TRP-2 expression in B16BL6 cells to 265.83 ± 12.99% in the FBS (2%)-alone-treated control. PSFAb significantly suppressed this increase only at 100 μg/mL to 178.94 ± 15.96% of the FBS (2%)-alone-treated control (Figure 4A,E).

### 2.5. PSFAb-Induced Changes in the Activation of MAPKs in B16BL6 Melanoma Cells

**Figure 5:**
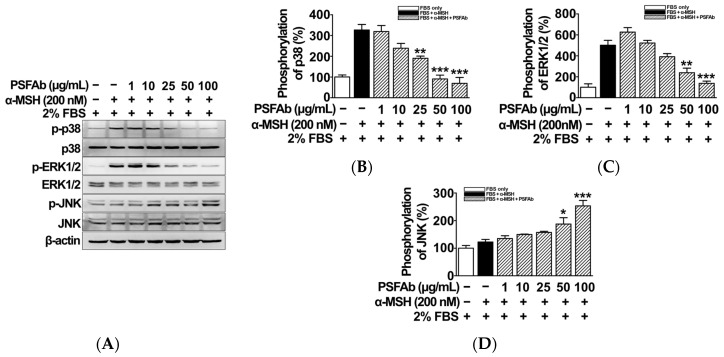
Effects of the *Persicaria senticosa* (Meisn.) H.Gross flower absolute on MAPK signaling pathways in B16BL6 cells. (**A**) Representative immunoblot data. The cells were exposed for 5 min to the *Persicaria senticosa* (Meisn.) H.Gross flower absolute (PSFAb; 1–100 μg/mL, prepared in the minimum essential medium containing 2% fetal bovine serum (FBS)) in the presence or absence of α-melanocyte-stimulating hormone (α-MSH; 200 nM). The protein extracts were subjected to immunoblot analysis using the indicated antibodies, as described in Section 4. In the treatment scheme shown in the figure, “+” and “−” indicate the presence and absence of the indicated treatment, respectively. The experimental groups were as follows: untreated control (2% FBS only), α-MSH alone (200 nM), and α-MSH plus PSFAb (1, 10, 25, 50, or 100 μg/mL). (**B**–**D**) Statistical graphs resulting from the quantitative analyses of the phosphorylation levels of p38 MAPK (**B**), ERK1/2 (**C**), and JNK (**D**). The untreated control (2% FBS only) served as the negative control, whereas α-MSH (200 nM) alone served as the positive control. The phosphorylation levels of each protein were normalized to the respective total protein and are presented as percentages relative to the untreated control (2% FBS only). β-Actin was used as a loading control to confirm equal protein loading. The results are presented as the mean ± standard error of the mean (SEM) from three independent biological replicates (*n* = 3). Statistical analysis was performed using one-way ANOVA followed by Tukey’s post hoc test. *: *p* < 0.05; **: *p* < 0.01; ***: *p* < 0.001 compared with cells treated with α-MSH alone. p-p38, phosphorylated p38 MAPK; p-ERK1/2, phosphorylated ERK1/2; p-JNK, phosphorylated JNK.

MAPKs are strongly associated with MITF regulation in the melanogenesis signaling pathway of melanocytes [9,11]. Given the findings observed above, namely the inhibitory effect of PSFAb on MITF expression in B16BL6 cells, this study further investigated how PSFAb (1–100 μg/mL) influences the activation of MAPKs, including p38 MAPK, ERK1/2, and JNK, using Western blot analysis. PSFAb (25–100 μg/mL) induced a significant reduction in α-MSH (200 nM)-stimulated p38 MAPK activation, which had been elevated to 326.48 ± 26.64% relative to the FBS (2%)-alone-treated control. The strongest inhibitory effect of PSFAb occurred at 100 μg/mL, with the activation level decreasing to 69.42 ± 28.47% of the FBS (2%)-alone-treated control (Figure 5A,B). Similarly, PSFAb at 50 and 100 μg/mL significantly attenuated ERK 1/2 activation induced by α-MSH (200 nM), which had increased to 510.21 ± 46.47% of the FBS (2%)-alone-treated control. This inhibitory effect was strongest at 100 μg/mL, with the level reduced to 137.00 ± 21.49% of the FBS (2%)-alone-treated control (Figure 5A,C). In contrast, the α-MSH (200 nM)-induced JNK activation level (122.41 ± 9.21% of the FBS (2%)-alone-treated control) was enhanced significantly by treatment with PSFAb at 50 and 100 μg/mL, reaching a maximum at 100 μg/mL (253.26 ± 19.80% of 2% FBS-alone-treated control in B16BL6 cells; Figure 5A,D).

### 2.6. PSFAb-Induced Changes in Melanophilin and Rab27a Involved in Melanosome Transport in B16BL6 Cells

**Figure 6:**
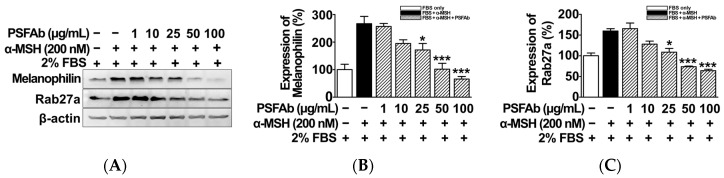
Effects of the *Persicaria senticosa* (Meisn.) H.Gross flower absolute on melanosome transport-related proteins in B16BL6 cells. (**A**) Representative immunoblot results. B16BL6 cells were treated with or without the *Persicaria senticosa* (Meisn.) H.Gross flower absolute (PSFAb; 1–100 μg/mL, prepared in the minimum essential medium containing 2% fetal bovine serum (FBS)) in the presence or absence of α-melanocyte-stimulating hormone (α-MSH; 200 nM) for 24 h. The protein extracts were analyzed by immunoblotting using the indicated antibodies. In the treatment scheme shown in the figure, “+” and “−” indicate the presence and absence of the indicated treatment, respectively. The experimental groups were as follows: untreated control (2% FBS only), α-MSH alone (200 nM), and α-MSH plus PSFAb (1, 10, 25, 50, or 100 μg/mL). (**B**,**C**) Statistical graphs obtained from quantitative analyses of melanophilin (**B**) and Rab27a (**C**) expression levels. The untreated control (2% FBS only) served as the negative control, whereas α-MSH (200 nM) alone served as the positive control. The expression levels of each protein were normalized to β-actin as a loading control, and the expression levels are presented as percentages relative to the untreated control (2% FBS only). The results were presented as the mean ± standard error of the mean (SEM) from three independent biological replicates (*n* = 3). Statistical analysis was performed using one-way ANOVA followed by Tukey’s post hoc test. *: *p* < 0.05; ***: *p* < 0.001 compared to the cells exposed to α-MSH alone.

Melanosome transport in melanocytes is mediated by the formation of a complex among melanophilin (Slac2-a), Rab27a, and myosin Va. Disruption of this protein complex has been reported to impair melanosome movement, leading to an abnormal melanin distribution and altered skin pigmentation [10,22]. B16BL6 cells were exposed to PSFAb at concentrations of 1–100 μg/mL, and immunoblotting was conducted to assess the influence of PSFAb on the expression of melanophilin and Rab27a. PSFAb reduced the α-MSH (200 nM)-stimulated increase in melanophilin expression in B16BL6 cells (266.80 ± 26.28% relative to the FBS (2%)-alone-treated control). Significant inhibition was detected at 25 to 100 μg/mL, with the strongest suppression at 100 μg/mL (65.75 ± 9.28% of the FBS (2%)-alone-treated control; Figure 6A,B). Similarly, PSFAb attenuated the α-MSH-upregulated expression of Rab27a (159.73 ± 5.53% compared to the FBS (2%)-alone-treated control). This reduction was significant at 25 to 100 μg/mL, with the largest inhibitory effect observed at 100 μg/mL (63.84 ± 3.41% of the FBS (2%)-alone-treated control; Figure 6A,C).

## 3. Discussion

This study reported that PSFAb, a flower-derived absolute fraction containing eight identified compounds, exerts broad suppressive effects on the melanogenic- and melanosome transport-related responses in B16BL6 melanoma cells. Considering the growing demand for safer skin-whitening agents, plant-derived materials have attracted attention as a central target owing to their relatively favorable safety profiles and diverse biological activities [9,23]. Accordingly, PSFAb was prepared and its skin pigmentation regulatory activities in B16BL6 melanoma cells, a widely used in vitro model due to their stable melanin-producing capacity, were evaluated [10,23].

In the present study, chemical composition analysis revealed that PSFAb contains eight identified compounds. Among these constituents, 1-docosanal (41.74%) and octadecanal (29.51%) were the predominant components of the extract. However, to the best of our knowledge, no studies have reported the biological effects of these substances on melanogenesis or skin pigmentation. Therefore, despite their high abundance in PSFAb, their individual contributions to the anti-melanogenic activity observed in the present study remain unclear. Additionally, linolenic acid accounted for 6.90% of the total composition. Previous studies have suggested that linolenic acid possesses various biological activities, including anti-inflammatory, neuroprotective, antidepressant and pigmentation-regulatory properties [24,25,26,27]. In particular, linolenic acid has been reported to inhibit melanogenesis by exhibiting an anti-proliferative effect on melanocytes and attenuating the melanin contents and tyrosinase activity in these cells [27,28,29]. In addition, linolenic acid has been shown to attenuate ultraviolet-induced hyperpigmentation in vivo [27]. These previous reports suggest that linolenic acid could be one of the potential contributors to the anti-melanogenic activity of PSFAb. However, given its relatively low abundance, the present study does not provide direct evidence that linolenic acid is the major active constituent responsible for the observed effects. Further studies are needed to determine the contribution of individual constituents, including 1-docosanal, octadecanal, and linolenic acid, to the anti-melanogenic activity of PSFAb. In addition, it will be important to clarify whether the observed effects result from a single active compound or from synergistic interactions among multiple components and to validate these findings in vivo. Furthermore, as PSFAb is a complex plant-derived extract, its chemical composition may be influenced by various biological and environmental factors, including the harvest season, geographical collection location, cultivation conditions, and batch-to-batch variation in plant materials. To facilitate the standardization and quality control of PSFAb, 1-tetracosanol (8.21%) may serve as a primary marker compound due to its reliable identification, relatively high abundance, and availability as an authentic reference standard, making it suitable for potential quantitative analysis. In addition, 1-docosanal and octadecanal, the two most abundant constituents of PSFAb, may serve as complementary marker compounds for chromatographic fingerprinting and evaluation of batch-to-batch chemical consistency. GC–MS profiling combined with the assessment of these marker compounds may provide a practical basis for monitoring the chemical profile and evaluating the consistency of PSFAb preparations. In the present study, PS flowers obtained from a single collection source and extraction batch were used to minimize potential variability. However, the stability of PSFAb during storage and the consistency of its chemical profile across different batches, seasons, and collection locations were not specifically evaluated. Future studies involving multiple PSFAb preparations with comprehensive chemical characterization will be required to establish the reproducibility and standardization of PSFAb composition and biological activity.

An evaluation of cellular proliferation revealed that PSFAb not only attenuated 2% serum-induced proliferation but also suppressed α-MSH-enhanced melanin production and tyrosinase activity in B16BL6 cells without affecting cell viability. These findings are consistent with previous reports suggesting that melanocyte proliferation is associated with melanogenesis and that attenuating proliferation can reduce melanin production [30,31]. However, the reduction in melanogenesis observed in the present study is unlikely to be explained solely by the inhibition of cellular proliferation. Although reduced proliferation may contribute to decreased melanin synthesis, the melanin content and tyrosinase activity measured in the present study were normalized to total protein content, thereby minimizing the influence of differences in cell number. Therefore, the inhibitory effect of PSFAb on melanogenesis is likely attributable not only to reduced cellular proliferation but also to the modulation of melanogenesis-related pathways. Although melanocyte proliferation and pigmentation are closely related biological processes, our findings suggest that PSFAb may exert regulatory effects beyond the inhibition of cell proliferation. Melanocyte functions, including their proliferation, differentiation, and survival mechanisms, fundamentally regulate skin pigmentation [31,32]. In particular, the absence of a significant reduction in cell viability suggests that the observed effect results from specific regulation of melanogenic processes rather than nonspecific cellular damage, supporting the potential biological relevance of PSFAb in pigmentation control.

In addition, an inhibitory effect of PSFAb on α-MSH-stimulated melanogenesis was also observed in B16BL6 cells. As α-MSH can activate melanogenic pathways, primarily by transcriptionally activating MITF [31,33], these results suggest interference at the regulatory level upstream of pigment synthesis. Similar inhibitory responses to those observed in this study have been reported for other plant-derived extracts that modulate α-MSH signaling cascades, highlighting the relevance of natural compounds in controlling melanogenesis [4,9].

Tyrosinase, TRP-1, and TRP-2, which are major enzymes driving melanogenesis [10], were examined to elucidate the inhibitory action of PSFAb on melanin production. In B16BL6 cells, PSFAb reduced tyrosinase expression, the enzyme responsible for the rate-limiting conversion of L-tyrosine to downstream melanin precursors [10,34], and attenuated tyrosinase activity associated with melanin production [31,33]. In parallel, downregulation of TRP-1 and TRP-2 expression was found in α-MSH-stimulated B16BL6 cells upon exposure to PSFAb.

MITF serves as the primary transcription factor that modulates the expression of critical melanogenic enzymes, specifically tyrosinase, TRP-1, and TRP-2 [10,21]. Therefore, MITF expression was examined. We found an inhibitory effect of PSFAb on the α-MSH-mediated increase in MITF levels within B16BL6 cells. As MITF functions as a master regulator of melanogenic gene expression [31,33], its downregulation led to a reduction in the expression of tyrosinase, TRP-1, and TRP-2 and consequently inhibited melanin production [33,35]. Thus, MITF downregulation provides a mechanistic explanation for the observed PSFAb-induced decrease in melanogenic enzyme expression and melanin production, suggesting that PSFAb likely exerts its effects at an upstream transcriptional regulatory level. The simultaneous inhibition of multiple melanogenic enzymes suggests that PSFAb may act on the melanogenic pathways through a multi-target mechanism rather than a single target. Similar multi-target inhibitory effects have been observed in other phytochemical studies [11,36], supporting the potential of PSFAb as a natural multi-target regulator of the melanogenic pathway. These findings suggest that PSFAb may contribute to reduced melanin production by simultaneously targeting and downregulating tyrosinase activity and the expression of key melanogenic molecules (tyrosinase, TRP-1, and TRP-2).

The upstream signaling pathways controlling MITF were evaluated, with particular attention to MAPK signaling. α-MSH stimulation typically activates the ERK1/2, JNK, and p38 MAPK pathways, which influence MITF expression and melanogenesis [36,37,38]. Nevertheless, the exact role of individual MAPK components in melanogenesis remains controversial because of inconsistent findings [11,36,37,38]. In addition, JNK signaling appears to have a less direct or inconsistent role in the regulation of MITF or melanogenesis [39,40]. Our results revealed that PSFAb exerted differential effects on MAPK signaling in B16BL6 cells, diminishing α-MSH-stimulated ERK1/2 and p38 phosphorylation while enhancing JNK activation. These findings suggest that PSFAb may not uniformly regulate MAPK signaling. Moreover, these distinct phosphorylation patterns may reflect differential modulation of MAPK signaling by PSFAb, as individual MAPK pathways can be differentially regulated by upstream signaling and cellular responses. However, the biological significance of these differential MAPK responses in PSFAb-mediated melanogenesis inhibition remains to be clarified. Given that MAPK signaling is involved in the regulation of MITF, we next examined MITF expression. MITF expression in B16BL6 cells was attenuated by the PSFAb treatment. The relationship between ERK1/2 signaling and MITF regulation remains controversial. Although ERK1/2 activation has been reported to promote degradation [38], other studies have suggested that alterations in ERK signaling, including both increased and decreased ERK activation, can suppress MITF expression and melanogenic protein levels, depending on the cellular context and upstream signaling mechanisms [11,41,42]. Therefore, our findings that PSFAb treatment reduced both ERK1/2 phosphorylation and MITF expression may reflect the context-dependent nature of ERK1/2-mediated regulation of melanogenesis rather than a simple linear relationship between ERK1/2 activity and MITF expression. However, the observed changes in ERK1/2, p38, and JNK phosphorylation should be interpreted with caution. Although the altered MAPK phosphorylation and reduced MITF expression observed suggest a possible association between MAPK modulation and MITF suppression, the present study does not establish a direct causal relationship between these events. Therefore, whether MAPK modulation directly contributes to MITF suppression via the anti-melanogenic effects of PSFAb requires further investigation.

In addition to melanogenesis regulation through melanogenic enzyme regulation, this study also examined melanosome transport-related proteins, which are considered another target in controlling pigmentation [14,15]. Melanosomes are melanin-containing organelles that move from melanocytes to the surrounding keratinocytes, contributing to epidermal pigmentation [12,31]. This transport is mediated by the coordinated action of three key proteins: myosin-Va, Rab27a, and melanophilin [14,15]. Disruption of any component in this complex can affect pigmentation by impairing melanin transfer [22,43]. In this study, the PSFAb treatment diminished the α-MSH-elevated expression of melanophilin and Rab27a in B16BL6 cells. This was accompanied by a decrease in melanin production, suggesting that PSFAb may modulate pigmentation by suppressing melanogenesis and downregulating melanosome transport-related proteins. Although PSFAb reduced the expression of melanosome transport-related proteins, including melanophilin and Rab27a, the present study did not directly evaluate intracellular melanosome distribution or transport activity. Therefore, these findings should be interpreted as molecular evidence suggesting a potential regulatory effect on melanosome transport rather than direct evidence of transport inhibition. Further studies using imaging-based approaches and functional transport assays will be required to determine whether PSFAb directly affects melanosome trafficking. Taken together, these findings suggest that PSFAb may exert anti-pigmentation effects by inhibiting melanogenesis and modulating the expression of melanosome transport-related proteins. However, the effective concentrations observed in this in vitro study cannot be directly translated to topical formulations because formulation stability, skin permeability, and bioavailability may influence the amount of PSFAb delivered to the target tissue. Further studies evaluating these factors are needed to determine its practical applicability.

## 4. Materials and Methods

### 4.1. Materials

Phosphate-buffered saline (PBS) and the minimum essential medium (MEM) were supplied by Welgene (Daegu, Republic of Korea). Gibco BRL (Gaithersburg, MD, USA) was the source for trypsin-ethylenediamine tetra-acetic acid (EDTA), penicillin/streptomycin (P/S), and fetal bovine serum (FBS). MilliporeSigma (St. Louis, MO, USA) served as the supplier for Triton X-100, bovine serum albumin, phenylmethylsulfonyl fluoride, L-DOPA, dimethyl sulfoxide (DMSO), and α-MSH. EZ-CyTox kits were supplied by DoGenBio (Seoul, Republic of Korea). For the cell proliferation enzyme-linked immunosorbent assay (ELISA), 5-bromo-2′-deoxyuridine (BrdU) kits were obtained from Roche (Mannheim, Germany). Antibodies against rabbit IgG, mouse IgG, MITF, phospho-ERK1/2, ERK1/2, phospho-JNK, JNK, phospho-p38 MAPK, and p38 MAPK were acquired from Cell Signaling Technology (Beverly, MA, USA). The melanophilin antibody was purchased from Proteintech (Wuhan, China). The Rab27a antibody was obtained from Santa Cruz Biotechnology (Dallas, TX, USA). Antibodies targeting tyrosinase, TRP-1, and TRP-2 were acquired from Abcam (Cambridge, UK). The anti-β-actin antibody was sourced from MilliporeSigma.

### 4.2. Preparation of the Persicaria senticosa (Meisn.) H.Gross Flower Absolute

On September 19, 2018, flowers of *Persicaria senticosa* (Meisn.) H.Gross (PS) were harvested from Hanaro Farm located in Songji-myeon, Jeollanam-do, Republic of Korea (34°23′00.4″ N 126°33′59.0″ E). Botanical identification was conducted by Dr. Jong-Cheol Yang at Baekdudaegan National Arboretum (Republic of Korea). A voucher specimen (No. PS-0001) was preserved in the Herbarium of the Korea Essential Oil Resource Research Institute and Korea Forest Plants Essential Oil Bank, Hoseo University. For the extraction process, 3.205 kg of PS flowers was treated with hexane (Samchun Chemicals, Pyeongtaek, Republic of Korea) for 1 h at room temperature (RT) according to a previously reported absolute extraction method [44,45]. After extraction, the extracts were concentrated under reduced pressure at 25 °C using a rotary evaporator (EYELA, Tokyo, Japan), resulting in dark yellow waxy concrete. This concrete was dissolved in 99.5% ethanol (Samchun Chemicals) and placed in cold storage (−20 °C) for 12 h. Following filtration via a sintered funnel (Daihan Scientific, Seoul, Republic of Korea) the filtrate was evaporated at 35 °C to obtain a light yellow, anhydrous wax fraction designated as the PS flower absolute (PSFAb; 2.21 g, corresponding to a 0.069% *w*/*w* yield based on the initial PS flower weight [3.205 kg]). The prepared PSFAb was stored at −80 °C in sealed containers (Bürkle GmbH, Bad Bellingen, Germany) until use and dissolved in DMSO immediately before subsequent assays. For each assay, stock solutions were prepared such that the final DMSO concentration was identical across all treatment groups, including the vehicle control (0.5% for the WST assay and 0.2% for all other cell-based assays).

### 4.3. Chemical Analysis of the Persicaria senticosa (Meisn.) H.Gross Flower Absolute and Identification of Its Components

To analyze the chemical compounds of PSFAb, GC-MS evaluation was carried out by the National Instrumentation Center for Environmental Management at Seoul National University (Seoul, Republic of Korea). For GC-MS, a Trace 1300 GC/TSQ 8000 (ThermoScientific, Waltham, MA, USA) equipped with a DB-5MS (60 m × 0.25 mm, 0.25 μm; Agilent Technologies, Inc., Santa Clara, CA, USA) was used. The injector was maintained at 250 °C, and helium was used as the carrier gas at a flow rate of 2.0 mL/min. Samples were injected at a split ratio of 1:20. The oven was initially kept at 35 °C for 2 min, then increased at 10 °C/min to 60 °C, at 2 °C/min to 120 °C (held for 5 min), and finally at 5 °C/min to 250 °C (held for 5 min). For GC-FID analysis, the detector temperature was set to 280 °C, with air, hydrogen, and make-up helium flows of 350 mL/min, 35 mL/min, and 40 mL/min, respectively. For GC-MS analysis, the interface temperature was 280 °C and the ion source temperature was 275 °C. Mass spectra were acquired in electron ionization (EI) mode with a scan time of 0.2 s over a mass range of *m*/*z* 35–300. Peak identification was done using retention indices (RIs) calculated with n-alkanes (C_7_–C_30_), and mass spectra were compared to those of standard compounds in the MS database of the National Institute of Standards and Technology (NIST) library.

### 4.4. Cell Culture

The B16BL6 murine melanoma cell line was supplied by the Intercellular Communication Network Lab (POSTECH, Pohang, Republic of Korea). Cell incubation was carried out using MEM containing 10% FBS and 1% P/S at 37 °C in a humidified atmosphere containing 95% air/5% CO_2_. For subsequent experiments, a cell confluency of 70–80% was maintained.

### 4.5. Cell Viability Assays

To assess cell viability, a WST (water-soluble tetrazolium salt) assay was performed utilizing the EZ-CyTox kits (DoGenBio). B16BL6 cells were seeded into 96-well plates at a density of 5 × 10^4^ cells/well and subsequently treated for 24 h with various concentrations of PSFAb in serum-free MEM supplemented with 0.5% DMSO. Next, 10 μL of the EZ-CyTox reagent was added to each well, followed by a 30 min incubation period at 37 °C. A multi-well plate reader (Synergy 2; Bio-Tek Instruments, Winooski, VT, USA) was then utilized to detect the absorbance at a wavelength of 450 nm.

### 4.6. Proliferation Assays

Assessment of cellular proliferation utilized the BrdU incorporation method using a commercially available kit from Roche (Indianapolis, IN, USA). B16BL6 cells (1.5 × 10^3^ cells/well) in a 96-well plate were treated with various concentrations of PSFAb prepared in MEM supplemented with 2% FBS and 0.2% DMSO. After 36 h of incubation, the cells were labeled with a 10 μM BrdU solution for an additional 12 h at 37 °C. Following cell fixation and DNA denaturation according to the manufacturer’s protocol, a monoclonal anti-BrdU antibody conjugated to peroxidase was applied to the cells for a 90 min incubation step at RT. The resulting luminescent signals were measured by utilizing a Synergy 2 microplate reader (Winooski, VT, USA) from Bio-Tek Instruments. Proliferative activities were calculated as relative percentages by normalizing luminescence values against those of untreated control cells.

### 4.7. Melanin Content Assay

B16BL6 cells (5 × 10^4^ cells/well) seeded into 6-well plates were incubated for 12 h. Subsequently, the cells were cultured for an additional 48 h at 37 °C in 2% FBS-supplemented MEM containing 0.2% DMSO, including or excluding specified concentrations of PSFAb, with or without 200 nM α-MSH. After a subsequent rinse with PBS, cell disruption was performed by treating the samples with a specific extraction solution that combined 1% Triton X-100 and 0.2 mM phenylmethylsulfonyl fluoride within a 0.1 M sodium phosphate-buffered base (pH 6.8). The resulting lysates underwent centrifugation (10,000× *g* for 15 min) to yield isolated cell pellets. These pellets were subsequently subjected to a 1 h heating step at 80 °C in 150 μL of 1 N NaOH with 10% DMSO to completely dissolve melanin. After repeated pipetting, the melanin content was quantified by measuring absorbance at 405 nm using a microplate reader (Synergy 2, Bio-Tek Instruments).

### 4.8. Tyrosinase Activity Assay

For determination of cellular tyrosinase activity, 5 × 10^4^ B16BL6 cells were plated in 6-well plates. Subsequent sample preparation, involving lysis and centrifugation, followed the aforementioned melanin content assay procedure. The assay mixture was prepared in a 96-well plate by combining 140 μL of the L-DOPA solution (2 mg/mL) with 60 μL of the isolated supernatant, followed by incubation at 37 °C for 60 min. The formation of dopachrome was quantified by measuring absorbance at 490 nm with a microplate reader (Synergy 2, Bio-Tek Instruments).

### 4.9. Immunoblotting

Cells were disrupted in RIPA (radioimmunoprecipitation assay) lysis buffer (Cell Signaling), and the lysates were clarified by centrifugation at 17,000× *g* for 15 min at 4 °C. The protein content within the collected supernatants were quantified using a detergent-compatible protein assay kit (Bio-Rad Laboratories; Hercules, CA, USA). Equal amounts of protein samples (30–80 μg per lane) were loaded onto 10% SDS-PAGE for electrophoretic separation. The proteins were then transferred to polyvinylidene fluoride membranes at 4 °C. The membranes were blocked with either 3% non-fat dry milk or 3% BSA at RT for 2 h, and then sequentially incubated with primary antibodies (1:1000–5000 dilution) and horseradish peroxidase-linked secondary antibodies. After incubation at RT for 1 h, detection of the target protein bands was performed using chemiluminescent reagents, and the images were acquired via an ATTO LuminoGraph II imaging system (Tokyo, Japan).

### 4.10. Statistical Analysis

GraphPad Prism (version 5.0), manufactured by GraphPad Software, Inc. (La Jolla, CA, USA), was utilized for all statistical analyses. Variations among multiple groups were determined by one-way analysis of variance (ANOVA) followed by Tukey’s post hoc test. The assumptions of normality and homogeneity of variance were considered before applying parametric statistical tests. Experimental results were expressed as the mean ± standard error of the mean (SEM) from independent biological replicates. A *p*-value of less than 0.05 was required to establish statistical significance.

## 5. Conclusions

PSFAb, which contained eight identified components, was evaluated for its effects on melanin pigmentation in B16BL6 cells. PSFAb suppressed serum-stimulated B16BL6 cell proliferation. In α-MSH-stimulated B16BL6 cells, PSFAb inhibited α-MSH-induced melanin synthesis and tyrosinase activity and downregulated key melanogenic proteins, including MITF, tyrosinase, TRP-1, and TRP-2. In addition, it modulated MAPK signaling by inhibiting p38 MAPK and ERK1/2 phosphorylation and promoting JNK phosphorylation. Furthermore, PSFAb downregulated the expression of proteins involved in melanosome transport, such as melanophilin and Rab27a. Overall, these findings suggest that PSFAb may regulate pigmentation-related responses by modulating the expression of melanogenesis- and melanosome transport-related proteins. Therefore, PSFAb may have potential utility as a bioactive material for the development of agents that regulate skin pigmentation and hyperpigmentation-related skin conditions. Nevertheless, further studies will be needed to isolate individual active constituents, validate their effects using advanced skin models, and confirm the functional effect of PSFAb on melanosome transport through imaging-based analyses. In addition, the safety profile of PSFAb, including skin irritation, toxicity, and tissue selectivity in relevant skin models, should be evaluated before further preclinical development.

## Figures and Tables

**Table 1:** Components in the *Persicaria senticosa* (Meisn.) H.Gross flower absolute.

No	Component Name	RT (min)		RI		Area (%)	CAS No.
			Observed	RSD (%)	Literature		
1	Tridecane	23.98	1300	0.00	1300	1.15	629-50-5
2	Methyl undecanoate	28.87	1426	0.00	1427	5.85	1731-86-8
3	2,4-Di-tert-butylphenol	31.55	1511	0.00	1511	0.17	96-76-4
4	Linolenic acid	45.08	2173	0.00	2162	6.90	463-40-1
5	Heneicosanol	48.46	2419	0.00	2401	6.47	15594-90-8
6	1-Tetracosanol	50.14	2455	0.00	2456	8.21	506-51-4
7	Octadecanal	50.25	2457	0.00	2400	29.51	638-66-4
8	1-Docosanal	51.74	2489	0.01	2430	41.74	57402-36-5
	Total Identified (%)					100.00	

RT: Retention time; RI: Retention index on a DB5-MS capillary column; RSD: Relative standard deviation; min: Minute. RSD (%) = (standard deviation (SD) of RI/mean of RI) × 100.

## Data Availability

The data presented in this study are provided in the article. Further inquiries can be directed to the corresponding author.

## Supplementary Materials

The following supporting information can be downloaded at: https://www.mdpi.com/article/10.3390/ph19071129/s1, Figure S1: Representative mass spectra of the compounds identified in the *Persicaria senticosa* (Meisn.) H.Gross flower absolute.

## Abbreviations

The following abbreviations are used in this manuscript:

PSFAb	Persicaria senticosa (Meisn.) H.Gross flower absolute
MITF	Microphthalmia-associated transcription factor
GC-MS	Gas chromatography–mass spectrometry
TRP-1	Tyrosinase-related protein-1
α-MSH	α-Melanocyte-stimulating hormone
TRP-2	Tyrosinase-related protein-2
MAPK	Mitogen-activated protein kinase
FBS	Fetal bovine serum
PBS	Phosphate-buffered saline
MEM	Minimum essential medium
P/S	Penicillin/streptomycin
BrdU	5-bromo-2′-deoxyuridine
RT	Room temperature or retention time
WST	Water-soluble tetrazolium salt
RIs	Retention indices
