# Supplementary material for: Phytochemical Composition and In Vitro Anti-Pigmentation Activity of Persicaria senticosa Flower Absolute: Potential Dual Inhibition of Melanogenesis and Melanosome Transport
Source: Pharmaceuticals (Basel). 2026 Jul 22;19(7):1129. doi: 10.3390/ph19071129 (PMC13414718; doi:10.3390/ph19071129)
Supplement: Supplementary file 1 [file pharmaceuticals-19-01129-s001.zip › pharmaceuticals-4405468-supplementary.pdf]

Supplementary Material

	Component name	RT (min)	RI		Area (%)	CAS No.
			Observed	Literature		
**A**	**Tridecane**	**23.98**	**1300**	**1300**	**1.15**	**629-50-5**

Mass spectrum

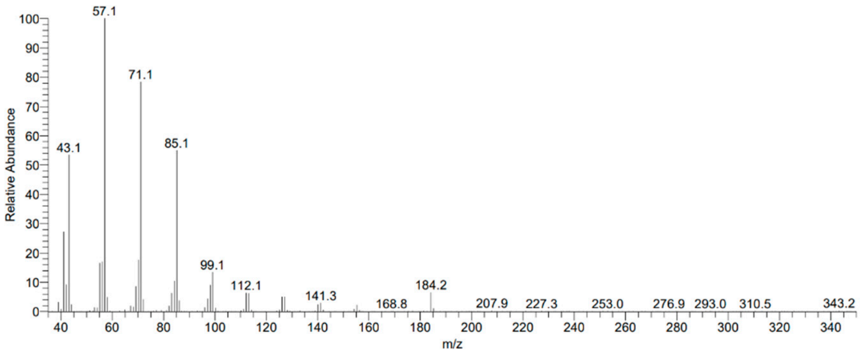

**B**	**Methyl undecanoate**	**28.87**	**1426**	**1427**	**5.85**	**1731-86-8**

Mass spectrum

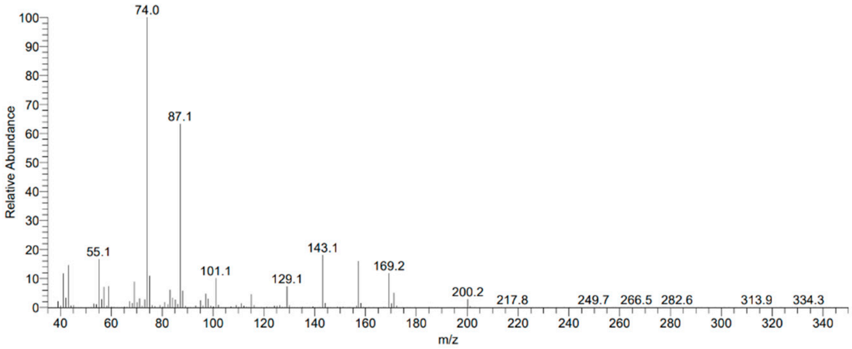

**C**	**2,4-Di-tert-butylphenol**	**31.55**	**1511**	**1511**	**0.17**	**96-76-4**

Mas spectrum

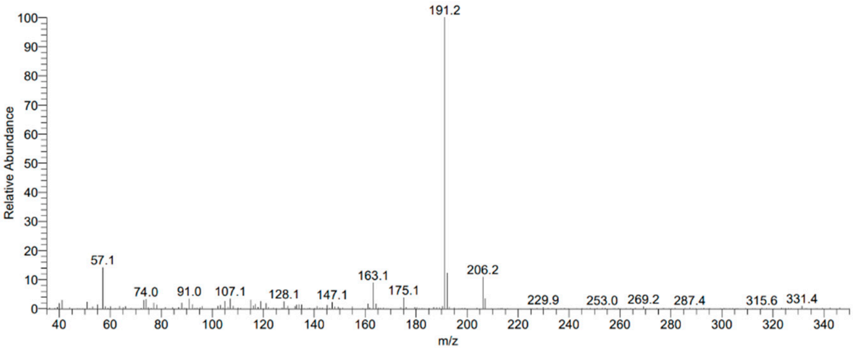

**D**	**Linolenic acid**	**45.08**	**2173**	**2162**	**6.90**	**463-40-1**

Mass spectrum

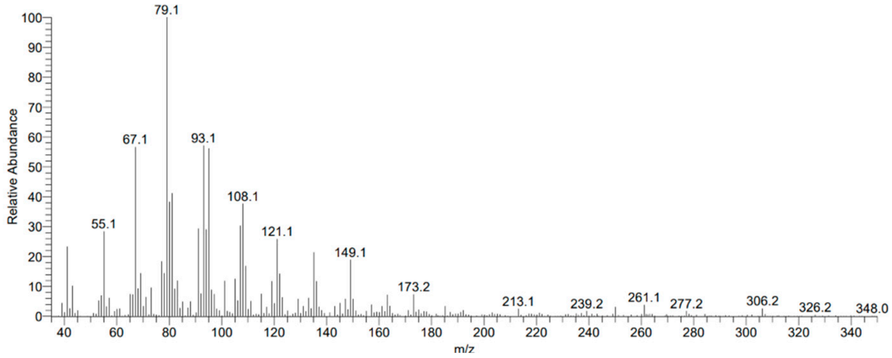

**E**	**Heneicosanol**	**48.46**	**2419**	**2401**	**6.47**	**15594-90-8**

Mass spectrum

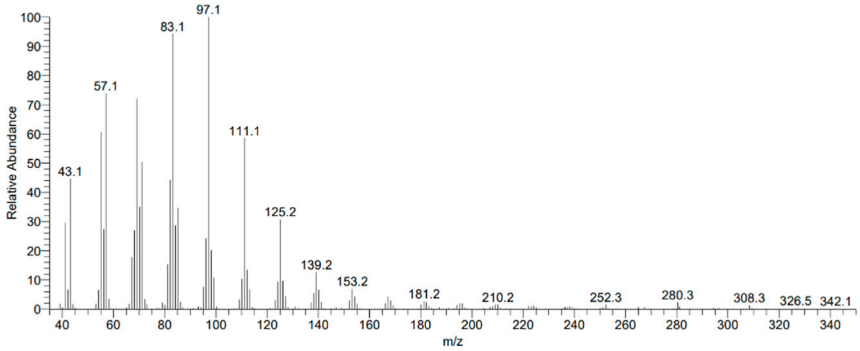

**F**	**1-Tetracosanol**	**50.14**	**2455**	**2456**	**8.21**	**506-51-4**

Mass spectrum

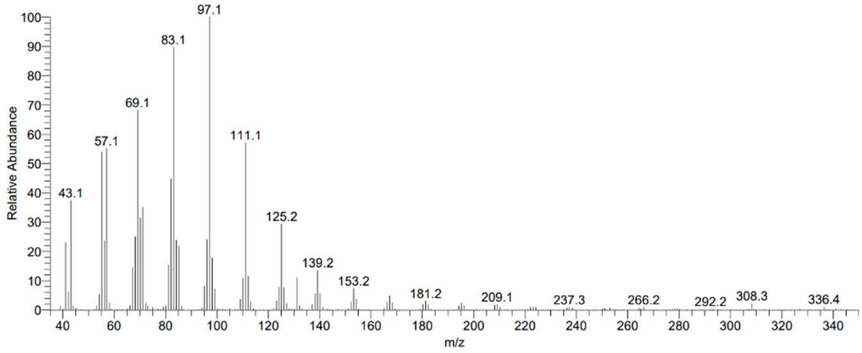

**G**	**Octadecanal**	**50.25**	**2457**	**2400**	**29.51**	**638-66-4**

Mass spectrum

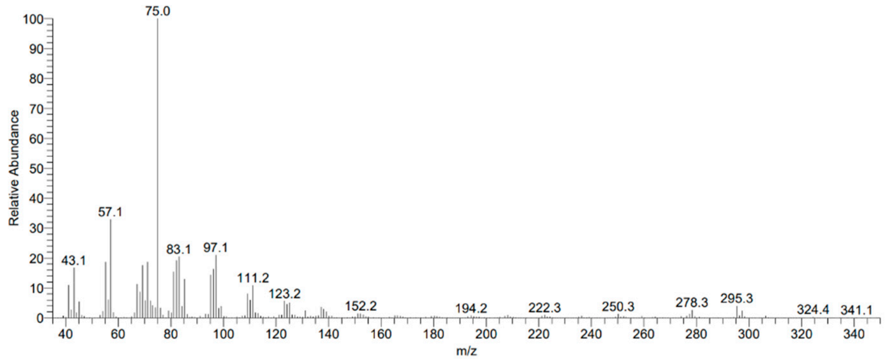

**H**	**1-Docosanal**	**51.74**	**2489**	**2430**	**41.74**	**57402-36-5**

Mas spectrum

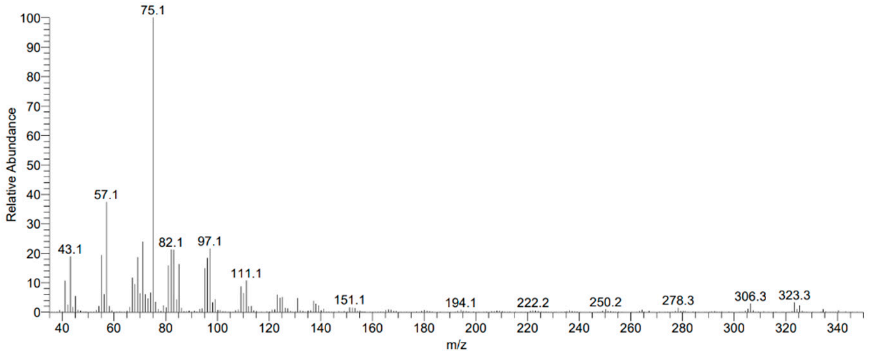

	**Total Identified (%)**	**100.00**

Supplementary Figure S1. Representative mass spectra of the compounds identified in the *Persicaria senticosa* (Meisn.) H.Gross flower absolute. RT: Retention time; RI: Retention indices on a DB5-MS capillary column; min: Minute.
